# Labial Length and Patient Symptomatology: Is There a Correlation?

**DOI:** 10.1093/asjof/ojae039

**Published:** 2024-06-11

**Authors:** Deepa Bhat, Ruth Tevlin, Kelsey Lipman, Francisco Canales, Heather Furnas

## Abstract

**Background:**

Labia minora length is used in classification systems and to determine labiaplasty candidacy, with shorter labia leading to nonsurgical recommendations.

**Objectives:**

The aim of the study was to investigate the correlation between labia length and symptomatology.

**Methods:**

Patients undergoing labiaplasty from January 2017 to May 2023 underwent chart review. Data collected included age, exposed, and total labia length. Patients completed a preoperative survey with possible scores from 0 to 13 to gauge complaints and symptoms.

**Results:**

Out of 50 charts with complete data, the average age was 34. Exposed labia lengths were 10.1 mm (right) and 11.4 mm (left); total lengths from sulcus to edge measured 32.0 mm (right) and 33.4 mm (left). Survey scores averaged 6.5 (range, 2-11) median of 7. The correlation between exposed labia length and symptoms yielded Pearson correlation coefficient values (*R*) of 0.25 for both right and left sides, with coefficient of determination (*r*^2^) values at 0.06. For total labia length, *R* values were 0.08 (right) and 0.06 (left), and *r*^2^ values were 0.007 (right) and 0.003 (left).

**Conclusions:**

The correlation between a patient's exposed and total labia length and reported symptomatology is weak. Patients with longer labia can experience few symptoms, just as those with shorter labia can have a high degree of symptomatology. Rather than use labia length as a primary factor determining labiaplasty candidacy, the focus should be on patient-reported symptoms.

**Level of Evidence: 2:**

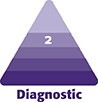

Patients seek reduction of their labia minora for disparate reasons, including discomfort, visibility in tight clothing, exposure in swimwear, dyspareunia, labial tugging and twisting, difficulties with hygiene, pain when engaging in sports, recurrent urinary tract infections, and psychological distress.^1-8^ Females seeking labiaplasty have lower sexual satisfaction, greater avoidance of sexual activity,^4^ and a poorer quality of life in comparison with those who do not seek labiaplasty. The goal of labiaplasty is to improve patient's symptoms by reducing the labia minora so that they do not project beyond the labia majora, tailoring the clitoral hood for proportionality, and improving symmetry and appearance.^8,9^ Labiaplasty's numbers have grown dramatically amid evidence that it is a safe procedure with low complication rates and high patient satisfaction rates, reflecting resolution of symptoms, improved genital perception, sexual function, and quality of life.^1,10-14^

Insurance companies and national health services often determine labiaplasty candidacy based on labia minora lengths greater than their defined “normal” range.^4^ Despite the lack of robust data linking labial length with patient symptomatology, those rejected for nonqualifying lengths are often referred for psychotherapy rather than being referred for a private-pay alternative.^15-20^ Labial length is also used in classification systems to determine labial hypertrophy, such as Franco's use of the length of the labia minora projecting beyond the labia majora: <2 cm (Type I); 2 to 4 cm (Type II); 4 to 6 cm (Type III); and >6 cm (Type IV).^16^

The aim of this project is to assess the correlation between labia length and symptomatology.

## METHODS

Patients requesting labiaplasty from January 2017 to May 2023 underwent chart review. This was a retrospective study using de-identified patient data that adhered to the guiding principles of the Declaration of Helsinki, and no aspect of the study impacted on patient care, obviating the need for Institutional Review Board approval. Variables examined included patient age, exposed labia length while standing, and total length from interlabial sulcus to edge while in a lithotomy position. All data were collected by a single surgeon (senior author).

Patients also completed a survey (Table 1) regarding symptoms, including twisting or discomfort in tight clothing, tugging, twisting, or pain during sexual intercourse, labia exposure in a bathing suit, visibility in tight clothing, feeling self-conscious about the appearance of their labia minora, diminished feelings of attractiveness to their partner, negative impact on self-esteem and/or intimacy, and restricting choice of underwear, bathing suits, and/or clothing. Possible scores ranged from 0 (no symptoms) to 13 (all symptoms). The authors’ own survey was used in the current absence of a validated survey covering a broad array of appearance-related and functional patient concerns.

**Table 1.** Summary of Patient Demographics and Measurements

	Age	Standing: length of exposed labium minus, relaxed, right (mm)	Standing: length of exposed labium minus, on stretch, right (mm)	% Increase relaxed to stretched, right	Standing: length of exposed labium minus, relaxed, left (mm)	Standing: length of exposed labium minus, on stretch, left (mm)	% Increase relaxed to stretched, left	Sulcus to labium minus edge, right (mm)	Sulcus to labium minus edge, left (mm)
Avg.	34.08	10.1	21.8	277	11.36	23.6	275	32	33.4
Range	16-63	0-25	0-44	125-1000	0-30	0-40	140-1150	6-65	10-62
Median	31	10	23	227	11	25	206	33	35

Descriptive statistics were calculated. The Pearson correlation coefficient (*R*), used to measure how strong a relationship is between 2 variables, as well as the coefficient of determination (*r*^2^), used to determine how many data points fall within the line formed by the linear regression equation, were also calculated.

## RESULTS

Out of the 212 labiaplasty patients reported during this time period, 50 charts had complete measurement data, the collection of which was impacted by factors, such as pandemic restrictions minimizing close contact. The average patient age was 34 (range, 16-63). The exposed labia length averaged 10.1 mm (range, 0-25 mm) on the right side and 11.4 mm (range, 0-30 mm) on the left. The median exposed labia length was 10 mm (right) and 11 mm (left). The total labia length averaged 32.0 mm (range, 6-65 mm) on the right and 33.4 mm (range, 10-62 mm) on the left. The median total labia length was 33 mm (right) and 35 mm (left) (Table 2).

**Table 2.** Survey Data

Survey question	Value	Percent
Uncomfortable twisting of the labia in tight clothing	30	60
Uncomfortable wearing tight clothing	26	52
Tugging during intercourse	32	64
Pain or discomfort with intercourse	19	38
Labia becomes exposed in a bathing suit	17	34
Visibility in yoga pants or tight clothing	24	48
Other	8	16
Self-conscious over the appearance of labia	44	88
Feel less attractive to partner	34	68
Negative impact of self-esteem	33	66
Negative impact on intimacy	28	56
Restricts clothing choices	23	46
Other	1	2

The survey scores ranged from 2 to 11, with a median score of 7 and an average score of 6.5 (Table 2). Thirty patients (60%) experienced uncomfortable twisting of the labia minora in tight jeans; 26 (52%) found tight jeans and pants uncomfortable to wear; 32 (64%) reported that the labia tugged or got in the way during sexual intercourse; 19 (38%) experienced pain or discomfort during sexual intercourse; 17 (34%) reported labia becoming exposed in a bathing suit; 24 (48%) noted that their labia minora were visible in tight exercise clothing or yoga pants; 44 (88%) patients felt self-conscious about their labia appearance, 34 (68%) felt less attractive to their partner, 33 (66%) associated their labia with a negative impact on their self-esteem 28 (56%) and on their intimacy; and 23 (46%) felt their labia minora restricted their choice of underwear, bathing suits, and/or clothing.

**Figure 1.**
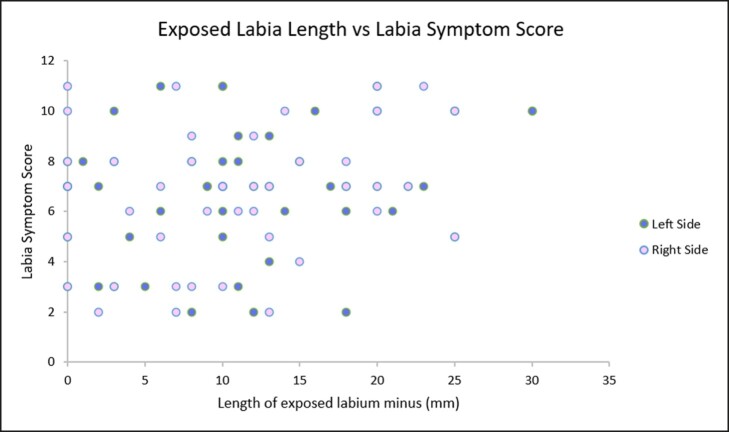
Relationship between exposed labia length and labia symptom score. This graph shows a weak correlation between exposed labia length and symptom scores. The coefficient of determination (*r*^2^) suggests that only 6% of patients demonstrated a linear increase in symptoms with exposed labia length.

**Figure 2.**
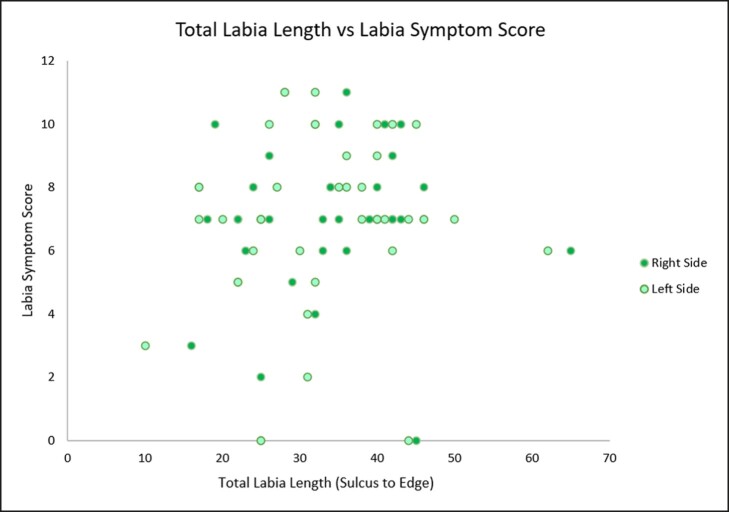
Relationship between total labia length and labia symptom score. This graph shows a very weak correlation between total labia length and symptom scores. The coefficient of determination (*r*^2^) suggests that very few (0.07% of right labia; 0.003% of left labia) demonstrate a linear increase with symptoms.

In a linear regression analysis comparing length of exposed labia with survey scores, the Pearson correlation coefficient (*R*) values were 0.25 (right) and 0.25 (left) and coefficient of determination (*r*^2^) values were 0.06 (right) and 0.06 (left) (Figure 1). In a similar linear regression analysis comparing total labia length with survey scores, the Pearson correlation coefficient (*R*) values were 0.08 (right) and 0.06 (left) and coefficient of determination (*r*^2^) values were 0.007 (right) and 0.003 (left) (Figure 2).

## DISCUSSION

Labia minora length is often used in classification systems and as the basis for surgical candidacy.^15-17^ The definition of labial elongation itself is widely debated. Although various classification systems qualify longer labial lengths as “hypertrophy,” implying abnormal anatomy,^15-17^ the majority of females seeking labiaplasty have labia that are considered “normal” in length.^6^ Ptotic or full labia majora can conceal elongated labia minora, and measurements themselves can be unreliable, as they vary with tension applied to the labia during measurement, intended or not. In the United Kingdom, patients with shorter labia are less likely to be referred to the National Health Service surgical services and more likely to be referred for counseling by their primary care physician^21^ or undergo private-pay labiaplasty compared with those with longer labia.^20^

Despite the assumption that greater labia lengths correlate with greater symptomatology, no significant relationship between labial measurements and genital perception or sexual function was demonstrated in a prospective cohort study.^18^ In addition, in a cross-sectional study of 200 premenopausal females, no correlation between labial length and self-reported labial symptoms was reported.^19^ Our study differs from the latter study in that these females were not seeking labiaplasty, whereas our patients were seeking surgical correction of their labia. In their study of 125 females in the United Kingdom, Veale et al also found no significant correlation between labia minora length and genital appearance satisfaction.^20^

Pearson correlation coefficient values for the relationship between exposed labia length and labia symptom scores as well as total labia length and symptom scores showed only a weak and very weak correlation, respectively, between length and symptoms. Similarly, the coefficient of determination (*r*^2^) suggests that only 6% of patients demonstrated a linear increase in symptoms with exposed labia length. Even fewer demonstrated a linear increase in symptoms with total labia length (0.07% of right labia; 0.003% of left labia). Clinically, this means that labia length is a poor indicator of the degree of symptomatology that patients experience. Notably, some patients with zero labial exposure reported symptom scores up to 11, and others with 18 mm of labial exposure reported symptom scores as low as 2.

The American College of Obstetricians and Gynecologists (ACOG) cites a lack of high-quality data supporting the effectiveness of genital cosmetic surgical procedures and proposes counseling and reassurance of anatomic normalcy rather than surgery to address patient concerns about the appearance of her external genitalia.^21^ ACOG further states that labiaplasty performed to alter sexual appearance or function is not medically indicated and poses substantial risk^22^ despite a growing body of evidence showing symptomatic improvement after labiaplasty, with low complications.^7,23-25^ The vast majority of patients seeking labiaplasty experience significant symptomatic relief of 90% or greater.^13,26,27^ In a study measuring symptomatology before and after surgery, nearly all (94%) were symptom free following labiaplasty.^13^

In the United States, labiaplasty procedures increased from 2020 to 2021 by 59%, to >19,000.^28^ Internationally, >171,000 procedures were reported in the 2021 International Society of Aesthetic Plastic Surgery annual report, a 20% increase from 2020.^29^ These numbers are likely underestimates, as labiaplasties are usually performed in an office procedure room and may not be reported.

The limitations of our study include a small sample size of 50 patients cared for by a single surgeon and the lack of a control group. Additionally, the administered labiaplasty symptoms survey covered common symptoms and complaints labiaplasty-seeking patients experience and was not a validated survey. Nonetheless, this study is one of very few demonstrating the weak correlation between labial length and symptomatology.^5^

## CONCLUSIONS

The correlation between a patient's exposed and total labia length and reported symptomatology is weak. Patients with longer labia can experience few symptoms, just as those with shorter labia can have a high degree of symptomatology. The nearly universal symptomatic relief most females experience after labiaplasty and the weak correlation of labia length with symptomatology underscore the importance of using patient-reported symptoms rather than labia length to determine labiaplasty candidacy.
